# Cognitive and behavioural therapy of voices for with patients intellectual disability: Two case reports

**DOI:** 10.1186/1744-859X-6-22

**Published:** 2007-08-19

**Authors:** Jérôme Favrod, Sabrina Linder, Sophie Pernier, Mario Navarro Chafloque

**Affiliations:** 1 Department of Psychiatry, University Hospital Center and University of Lausanne, Site de Cery, CH-1008 Prilly, Switzerland

## Abstract

**Background:**

Two case studies are presented to examine how cognitive behavioural therapy (CBT) of auditory hallucinations can be fitted to mild and moderate intellectual disability.

**Methods:**

A 38-year-old female patient with mild intellectual disability and a 44-year-old male patient with moderate intellectual disability, both suffering from persistent auditory hallucinations, were treated with CBT. Patients were assessed on beliefs about their voices and their inappropriate coping behaviour to them. The traditional CBT techniques were modified to reduce the emphasis placed on cognitive abilities. Verbal strategies were replaced by more concrete tasks using roleplaying, figurines and touch and feel experimentation.

**Results:**

Both patients improved on selected variables. They both gradually managed to reduce the power they attributed to the voice after the introduction of the therapy, and maintained their progress at follow-up. Their inappropriate behaviour consecutive to the belief about voices diminished in both cases.

**Conclusion:**

These two case studies illustrate the feasibility of CBT for psychotic symptoms with intellectually disabled people, but need to be confirmed by more stringent studies.

## Background

Lifetime prevalence of psychosis is higher among people with mild intellectual disability (ID) than in the general population [1-4]. However, few studies have assessed the effectiveness of psychological treatments [5] within this population.

Compared to patients without mental retardation, patients with mild mental retardation display different patterns in expressing psychiatric symptoms. For example, psychotic symptoms frequently involve hallucinations without delusion and less frequently delusion alone. Patients with mental retardation present more symptoms involving actions rather than thoughts and have a tendency to display more symptoms directed against others and less against themselves [6]. It is generally acknowledged that auditory hallucinations can reliably be detected among people with mild retardation [4,7,8].

Cognitive and behavioural therapies (CBT) of psychotic symptoms have been developed with the aim to reduce the distress associated with delusional ideas and hallucinations, as well as to improve the patients' coping ability. A recent meta-analysis dealing with CBT of positive symptoms of illnesses within the schizophrenia spectrum disorders is conclusive about the utility of CBT for the treatment of psychoses [9].

Many studies have debated the possibility to use CBT for patients with mental disability. Some suggest that the patient's poor verbal and abstract thinking abilities constitute an obstacle to the application of this method of treatment, whereas others underline the possibility of adapting the CBT to the patients' cognitive abilities [see [10] for review]. Haddock et al. [11] recommended some straightforward modifications concerning the practical application of CBT rather than to the theoretical foundation of the approach itself. The adaptations were: slower pace, adaptation of explanatory materials, involvement of the carers, and careful assessment of the participant's abilities to make thought-feeling-behaviour links. In order to replicate these results, two single case studies of CBT of voices with patients with mild and moderate mental retardation are described in this paper.

## Methods

### Setting

Both patients have been treated by the team of the outpatient liaison psychiatry consultation for the intellectually disabled of the Community psychiatry service of the Department of psychiatry in Lausanne, Switzerland.

### Subjects

Patient 1 is a 38-year-old female, living in a sheltered apartment. She suffers from daily auditory hallucinations and mild ID. Voices have said: "It's me. I'm coming!" According to her, the voice is very real and she attributes it to her previous boyfriend who left her 2 years ago without any word of explanation. When she hears the voice, she calls her previous boyfriend and insults him. She has been hospitalised since she started yelling at him alone in her room and smashing objects against the wall. She has been treated with 150 mg of quietiapine per day for the past 2 years.

Patient 2 is a 44-year-old male with a moderate ID. He is living in a unit of a specialized institution. The patient is hearing voices that say that he is lazy and doesn't work enough. They threaten him with sanctions or death if he doesn't comply with the orders. In reaction to his voices, the patient is stressed and sweats. This accelerated pace is dangerous when he is working or out in public because he is liable to injure himself at work or, for example, forget elementary safety pedestrian rules when on the street. He has been treated with 500 mg of clozapine daily for the past 5 years.

### Measures

For patient 1, the Beliefs about Voices Questionnaire – revised (BAVQ-R) has been used as a repeated measure [12]. The BAVQ-R is a 35-item self-report instrument that measures how people perceive and respond to their verbal auditory hallucinations. Frequency of voices was assessed on a 7-point scale ranging from "continuously" to "no voice this past week". As a more objective dependant measure, monthly portable phone bills have been used.

For patient 2, a more rudimentary scale was used as the patient answered the BAVQ-R without consistency. The patient had to quote the power of the voice on a 10-point analogical scale ranging from: "voices are very powerful" to "voices are very weak". Agitation was measured on a 10-point scale with his key social worker. Patient 2 was assessed monthly.

### Treatment

The basic intervention followed the Haddock et al. [11] recommendations concerning the practical application of CBT. The following supplementary modifications were made to the intervention. Progressive relaxation techniques were taught to reduce anxiety about psychotic symptoms. Concrete exercises showing how the brain can be tricked have been used in order to normalize psychotic symptoms. Exercises included optical and tactile [13,14] illusions that can directly be experienced by the patient.

Strategies to cope with voices were tried, practiced and fitted to patient environment. For example, it appeared for patient 2 that humming was effective and acceptable when walking. The sheltered workshop coach accepted this strategy. After 2 weeks, colleagues complained about the patient singing loudly and out of tune. The strategy was consequently abandoned at work and replaced unsuccessfully by listening to music on a personal stereo. Finally, the patient and the therapist together recorded an answer to the voices developed during the roleplaying. The patient was then trained to use it with his personal stereo when hearing voices. The strategy was judged effective by the patient and the sheltered workshop coach.

As an alternative to the traditional verbal challenge of evidence supporting beliefs about voices, more concrete techniques were used to reduce the emphasis on abstract thinking. Roleplays were used in which patients have to respond to the voices and disobey their orders. To start with, the patient takes the place of the voice and the therapist answers to model effective responses, and then the roles are reversed. The patients' theories about voices were discussed with figurines. Patient 1 thought she had better hearing than other people, which is why she could hear her former boyfriend when others could not. Figurines were helpful to test and challenge the belief. For example, in order to test her theory about her hearing, the patient had to choose two figurines, one representing herself another the therapist. They were placed on a table representing the office. A third figurine representing the previous boyfriend was placed three meters away to symbolize his place of work. The patient was asked to explain how she could hear her boyfriend's voice and why the therapist could not. She defended the idea that was the consequence of her better hearing. Then, two new figurines were selected to characterize two people talking together between the office and the workplace of the previous boyfriend. The patient accepted the idea that two people might be talking at a distance. The therapist asked if the patient could hear these people talking as well. The patient had to admit that she couldn't, and realised that her theory was not a valid explanation of the phenomena.

Concrete reality tests were constructed and tested with the patient. The tests included the use of noise protection devices and an audio recorder to test if the voices came from inside or from an outside physical source. More specifically to test the hearing of patient 1, exercises consisting of listening to people on the street were used as reinforcement of the challenge of the belief practiced in the office. Patient 2 had to disobey to orders given by the voices in the presence of the therapist and observe if the threats the voices made were carried out.

## Results

**Figure 1:**
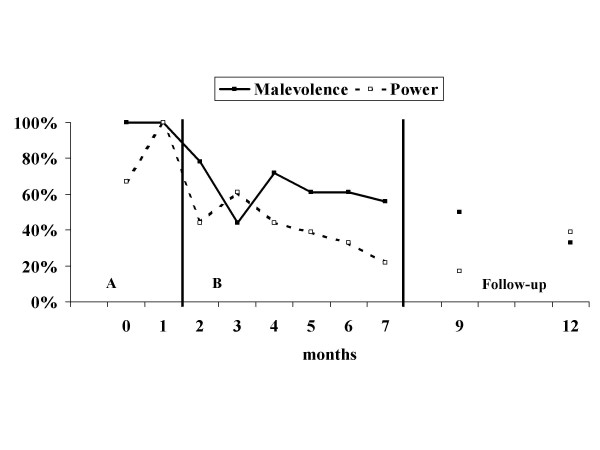
**BAVQ-R for patient 1**. BAVQ-R = Belief about voices questionnaire – Revised, malevolence and power scales. A, baseline; B, intervention.

**Figure 2:**
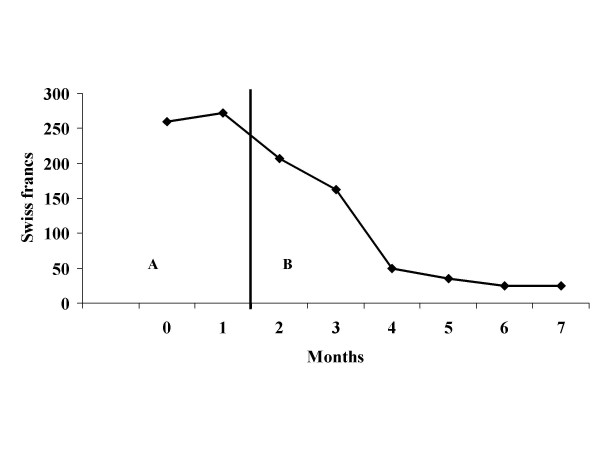
**Patient 1's phone bills**. A, baseline; B, intervention.

Figures 1 and 2 show the results for patient 1. Beliefs about voices on the malevolence scale decrease with the application of the intervention, and results were maintained at follow-up points. The power scale follows the same curve with an increase at the second follow-up. The frequency of voices changes from several times a day during the pre-test phase to several times a week at the end of therapy and the follow-up phase. Phone bills have been reduced radically following the introduction of the therapy. As patient 1 changed her mobile phone contract's subscription rate and type, follow-up was stopped at the eighth month. Reduction on the point scales was maintained.

**Figure 3:**
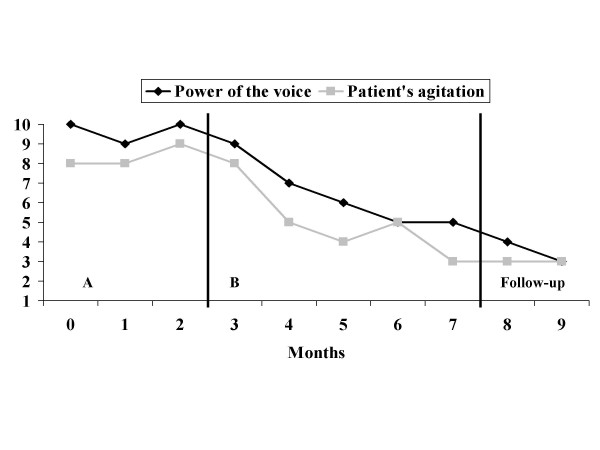
**Results for patient 2**. A, baseline; B, intervention.

Figure 3 shows the results for patient 2. Graphs show that the patient reduced the power that he attributed to the voices as well as his level of agitation as assessed by his team. Progress was maintained at the 2-month follow-up. Contact with his sheltered workshop coach and the patient indicates that progress had been maintained at the 2-year follow-up.

## Discussion

These two case studies indicate that CBT of voices for patients with ID can be applied in clinical routine. The application of the intervention seems to affect the dependant variables directly. Despite a global amelioration, patient 1 showed an increase on the power scale at 12 months during follow-up which remained however below the baseline. The belief regarding malevolence of the voice is continually improving. Patient 2 shows a continuing improvement. Informal follow-up meetings during a 2-year period did not show evidence of any relapses or exacerbations leading to behavioural problems in either patient.

However, in order to circumvent abstract thinking limitations, most of the cognitive aspects of CBT have been modified into more behavioural ones. The adaptations in the delivery of CBT have been greater than those recommended by Haddock et al. [11]. It should be considered whether the foundations of the approach have been radically altered. In our opinion, the spirit of CBT of voices has been kept, but transformed in a more physical way using behavioural components to challenge beliefs about voices.

## Conclusion

No definite conclusions can be drawn from these isolated case studies. Absence of control threatens the validity of the results. Patient 1's baseline has been limited at two points measurement because she was actively expressing suffering due to her voices and required quick intervention. Patient 2 lived in a sheltered environment and necessitated a more complex behavioural analysis, allowing a longer baseline. In the absence of controls, progress can be attributed to the single psychological attention given to the patients. However, patients with mild to moderate ID do not usually accede to specialized therapists in CBT of psychotic symptoms and our team do not meet a sufficient number of patients with dual diagnosis of psychotic symptoms and concomitant ID to lead a controlled study.

## Competing interests

The author(s) declare that they have no competing interests.

## Authors' contributions

JF led the two therapies and gathered the data. JF, SL, SP and MNC drafted the manuscript. All authors read and approved the final manuscript.
